# Supplementary material for: Content-rich biological network constructed by mining PubMed abstracts
Source: BMC Bioinformatics. 2004 Oct 8;5:147. doi: 10.1186/1471-2105-5-147 (PMC528731; doi:10.1186/1471-2105-5-147)
Supplement: Additional File 2 — The original results of the above study (non-essential files are deleted to keep the file size under the limit set by BMC bioinformatics). [file 1471-2105-5-147-S2.bz2 › chilibotAdditionalFile2/dip50/704ID10734142E18041/html/MUTL_MUTS.html]

 


 **MUTL** and **MUTS** 
  
Found 186 abstracts in PubMed, retrieved 50.  
 

 What does Google say? 
 PDF only 
| .edu only 

---

**Interactive relationship** (e.g. stimulation, inhibition, etc)

**Neutral relationship**- Interaction of Escherichia coli  **MutS**  and  **MutL**  at a DNA mismatch.  Ref: 11371566 J Biol Chem, 2001
- Fluctuation tests with yshD knock out mutants did not indicate any role for the protein in preventing the accumulation of spontaneous forward mutations to RifR, nor was any functional interaction with  **MutS**  or  **MutL**  suggested in fluctuation experiments with mutants lacking combinations of the three genes.  Ref: 11254128 Mol Gen Genet, 2001
- Likewise, many prokaryotes could operate similarly, independently of  **MutL**  by encoding a fused  **MutS**  Smr MutS2 protein.  Ref: 10973051 Trends Biochem Sci, 2000
- Such a higher order  **MutS**  complex productively interacts with  **MutL**  in ATP hydrolyzing conditions and generates a specific ternary complex, which might communicate with MutH.  Ref: 11888282 Biochemistry, 2002
- Human mismatch repair protein  **MutL**  homologue 1 MLH1 interacts with Escherichia coli  **MutL**  and  **MutS**  in vivo and in vitro a simple genetic system to assay MLH1 function.  Ref: 12513688 Biochem J, 2003
- The pathway in eukaryotes consists of the DNA binding components, which are the homologs of the bacterial  **MutS**  protein MSH 2 6, and the  **MutL**  homologs, which bind to the  **MutS**  homologs and are essential for the repair process.  Ref: 12091911 Nat Genet, 2002
- Both pathways require the formation of a  **MutS**  homolog complex which interacts with a second heterodimer, composed of two  **MutL**  homologs.  Ref: 12836196 Med Sci Paris , 2003
- **MutS**  initiates repair by binding to the mismatch, and activates together with  **MutL**  the MutH endonuclease, which incises at hemimethylated dam sites and thereby mediates strand discrimination.  Ref: 11920679 J Cell Physiol, 2002
- In one of these cases, in a coral mitochondrial genome, a gene is present that encodes a  **MutS**  protein fused to an HNH nicking endonuclease, potentially eliminating the requirement for  **MutL** .  Ref: 10973051 Trends Biochem Sci, 2000
- **MutL**  related proteins were also required for mutation avoidance in stationary phase cells, but to a lesser extent than  **MutS**  homologues.  Ref: 12491007 Curr Genet, 2002
- However, many genomes lack  **MutL**  but possess  **MutS** .  Ref: 10973051 Trends Biochem Sci, 2000
- In other bacteria, the requirement for sequence identity during recombination is partly due to the actions of the mismatch repair system, a key component of which,  **MutS** , recognizes mismatched bases in heteroduplex DNA and, along with  **MutL** , blocks strand exchange.  Ref: 11698371 J Bacteriol, 2001

**Non-interactive relationship** (e.g. studied together, co-existance, homology, etc.)

- As compared to native  **MutS** , the ability of MutS800 to promote  **MutL**  dependent activation of MutH is substantially reduced.  Ref: 12829697 J Biol Chem< MedlineTA>J Biol Chem, 2001
- The average percentages of positively stained cells for human  **MutL**  homolog 1 hMLH1, human  **MutS**  homolog 2 hMSH2, and human  **MutS**  homolog 6 hMSH6 in RGP CMM 75.6 3.4%, 67.20 7.71%, and 76.6 2.1%, respectively were reduced compared with benign nevi.  Ref: 12112321 Mol Carcinog, 2002
- When  **MutL**  is present, it associates with  **MutS**  and prevents ATP dependent migration away from the mismatch in a manner that is dependent on the length of the heteroduplex.  Ref: 11371566 J Biol Chem, 2001
- These effects parallel the  **MutL**  and  **MutS**  binding and ATP binding hydrolysis activities of the mutated proteins.  Ref: 12513688 Biochem J, 2003
- The products of  **mutS**  and  **mutL** , which are required for adenine methylation directed mismatch repair MMR, enhance VSP repair.  Ref: 11591694 J Bacteriol, 2001
- However, several  **MutS**  like and  **MutL**  like proteins also participate in meiotic recombination.  Ref: 12095912 Hum Mol Genet, 2002
- Following a common mismatch recognition step effected by  **MutS**  homologous Msh proteins, homologs of the bacterial  **MutL**  ATPase predominantly the Mlh1p Pms1p heterodimer in yeast couple mismatch recognition to the appropriate downstream processing steps.  Ref: 12454061 Genetics, 2002
- Mismatch repair in many organisms depends on three proteins the mismatch recognition protein  **MutS** , a nicking endonuclease MutH, and  **MutL** , which acts as a scaffold between these.  Ref: 10973051 Trends Biochem Sci, 2000
- Protein components responsible for these reactions are highly conserved through evolution, and homologs of bacterial  **MutS**  and  **MutL** , which are key players in the initiation steps of both the strand specific mismatch correction and MMR dependent apoptotic signaling, have been identified in human cells.  Ref: 12700127 Front Biosci, 2003
- Transcription of  **mutS**  and  **mutL**  homologous genes during meiosis in Saccharomyces cerevisiae and identification of a regulatory cis element for meiotic induction of MSH2.  Ref: 11523800 Mol Genet Genomics, 2001
- **MutS**  and  **MutL**  are both required to activate downstream events in DNA mismatch repair.  Ref: 11371566 J Biol Chem, 2001
- Exonuclease 1 Exo1 is a 5 3 exonuclease that interacts with  **MutS**  and  **MutL**  homologs and has been implicated in the excision step of DNA mismatch repair.  Ref: 12629043 Genes Dev, 2003
- iv defects in  **mutL**  or  **mutS**  appeared to have a stronger effect on growth dependent mutations than on adaptive mutations.  Ref: 11733076 Mutat Res, 2001
- We analysed the expression of two mismatch repair proteins, human  **mutL**  homologue 1 hMLH1 and human  **mutS**  homologue 2 hMSH2, and evaluated the frequency of microsatellite instability with 10 mononucleotide and two dinucleotide repeat sequences, in 39 paired tumour normal DNA samples obtained from 17 testicular and two mediastinal germ cell tumours.  Ref: 11719586 Mol Hum Reprod, 2001
- Multiple  **MutS**  and  **MutL**  homologues exist in eukaryotes, which play different roles in the mismatch repair MMR pathway or in recombination.  Ref: 11920679 J Cell Physiol, 2002
- At a low dose, dP is much more mutagenic in mismatch repair defective mutH,  **mutL** , and  **mutS**  strains than in a wild type strain.  Ref: 12196386 Genetics, 2002
- The mismatch repair MMR system plays a central role in maintaining genetic stability and requires evolutionarily conserved protein factors, including  **MutS**  and  **MutL**  homologs.  Ref: 12095912 Hum Mol Genet, 2002
- We also show that interaction between Vsr and  **MutL**  inhibits the ability of  **MutL**  to dimerize, to interact with  **MutS**  and MutH and to mediate a previously unknown interaction between  **MutS**  and MutH.  Ref: 11585365 Mutat Res, 2001
- The human mismatch repair hMMR system consists of members of three super families, namely MutH,  **MutL**  and  **MutS** .  Ref: 12578652 Zhongguo Shi Yan Xue Ye Xue Za Zhi< MedlineTA>Zhongguo Shi Yan Xue Ye Xue Za Zhi, 2001
- Insertional inactivation of  **mutS**  and  **mutL**  in the slow strain led to 500 and 250 fold increases in hmbR switching frequency respectively.  Ref: 11359570 Mol Microbiol, 2001
- The introduction of mismatches by growth in 2 aminopurine caused a replication dependent increase in the number of cells with  **MutS**  and  **MutL**  foci.  Ref: 11779496 Mol Cell, 2001
- The rate and extent of mismatch provoked cleavage at hemimethylated GATC sites by MutH in the presence of  **MutS** ,  **MutL** , and ATP are the same whether the mismatch and GATC sites are in cis or in trans.  Ref: 11371566 J Biol Chem, 2001
- The mutator phenotype was due to the ability of hMLH1 protein to interact with bacterial  **MutL**  and  **MutS**  proteins, thereby interfering with the formation of complexes between MMR proteins and mismatched DNA.  Ref: 12513688 Biochem J, 2003
- In addition, mutation analysis was performed for Transforming growth beta type II receptor TGF beta R II, bcl 2 associated X protein BAX, Insulin like growth factor II receptor IGF II R, human  **MutS**  homolog 3 hMSH3, human  **MutS**  homolog 6 hMSH6, human  **MutS**  homolog 2 hMSH2, and human  **MutL**  homolog 1 hMLH1 genes.  Ref: 10962455 J Surg Oncol, 2000
- An unsolved problem in E. coli mismatch repair is how the  **MutS**   **MutL**  complex communicates positional information of a mismatch to MutH.  Ref: 11888282 Biochemistry, 2002
- Seven out of the 11 mutator strains were found to be defective in the MMR system four  **mutS** , two  **mutL**  and one uvrD.  Ref: 11952911 Mol Microbiol, 2002
- Finally, every mutation that abolishes mismatch repair is deficient in blocking homeologous recombination, suggesting that mismatch repair and prevention of homeologous recombination use the same  **MutS**   **MutL**  complexes for signaling in E. coli.  Ref: 12606120 DNA Repair Amst , 2003
- We examined the rate of dissociation of  **MutS**  from a mismatch using linear heteroduplex DNAs or heteroduplexes blocked at one or both ends by four way DNA junctions in the presence and absence of  **MutL** .  Ref: 11371566 J Biol Chem, 2001
- We have used the recently determined crystal structures of Escherichia coli E. coli  **MutS** ,  **MutL**  and MutH to guide construction of 47 amino acid substitutions in these proteins and analyzed their behavior in mismatch repair and recombination in vitro and in vivo.  Ref: 12606120 DNA Repair Amst , 2003
- In four cases three  **mutS**  and one  **mutL** , the genes contained frameshift mutations.  Ref: 11952911 Mol Microbiol, 2002
- In eukaryotic cells, DNA mismatch repair is initiated by a conserved family of  **MutS**  Msh and  **MutL**  Mlh homolog proteins.  Ref: 12529393 Mol Cell Biol, 2003
- The  **mutS**  and  **mutL**  genes, which are contiguous in the L. monocytogenes chromosome, were identified after in silico analysis.  Ref: 11994166 Mol Microbiol, 2002
- The mismatch repair system  **mutS** ,  **mutL**  and uvrD genes in Pseudomonas aeruginosa molecular characterization of naturally occurring mutants.  Ref: 11952911 Mol Microbiol, 2002
- The growth defect and filamentation phenotypes associated with 2 aminopurine exposure are effectively suppressed by null mutations in mutH,  **mutL** ,  **mutS** , or uvrD mutU, which encode activities that act upstream of the four exonucleases in the mechanism for the methyl directed reaction that has been proposed based on in vitro studies.  Ref: 11381137 Proc Natl Acad Sci U S A, 2001
- In eukaryotes, homologs of the Escherichia coli  **MutS**  and  **MutL**  proteins are crucial for both meiotic recombination and post replicative DNA mismatch repair.  Ref: 12836196 Med Sci Paris , 2003
- These results suggest that a  **MutS**   **MutL**  complex in the vicinity of a mismatch is involved in activating MutH.  Ref: 11371566 J Biol Chem, 2001
- We also describe the alterations in the  **mutS** ,  **mutL**  and uvrD genes responsible for the mutator phenotype of hypermutable P. aeruginosa strains isolated from CF patients.  Ref: 11952911 Mol Microbiol, 2002
- Strand specificity and repair efficiency are largely independent of the GATC methylation state of the DNA and presence of the products of mismatch repair genes mutH,  **mutL** , and  **mutS** .  Ref: 12692132 J Biol Chem< MedlineTA>J Biol Chem, 2002
- MutH,  **MutL**  and  **MutS**  are essential components of the mismatch repair system in Escherichia coli.  Ref: 11934505 FEMS Microbiol Lett, 2002
- In eukaryotes, there are multiple homologs of the key bacterial  **MutS**  and  **MutL**  MMR proteins, and these homologs form heterodimers that have discrete roles in MMR related processes.  Ref: 11092832 Annu Rev Genet, 2000
- We have analysed the levels of mRNA transcripts of the  **mutS**  and  **mutL**  homologous genes of the yeast Saccharomyces cerevisiae during the course of meiosis, by quantitative RT PCR.  Ref: 11523800 Mol Genet Genomics, 2001
- The results shown here indicate that the putative P. aeruginosa  **mutS** ,  **mutL**  and uvrD genes are mutator genes.  Ref: 11952911 Mol Microbiol, 2002
- To address these questions, mice with inactivating mutations in all the known  **mutS**  and  **mutL**  homologs have been generated.  Ref: 12114115 Trends Mol Med, 2002
- **MutS**  hMSH2 and hMSH6 and  **MutL**  hMLH1, hPMS2, and hPMS1 DNA MMR protein and RNA levels were determined.  Ref: 12145807 Gastroenterology, 2002
- RESULTS All cell lines examined showed decreased levels of  **MutS**  and  **MutL**  DNA MMR proteins in a dose dependent manner after coculture with H. pylori strains.  Ref: 12145807 Gastroenterology, 2002
- To investigate the effects on PV rates of mutations in genes for mismatch repair MMR, insertion deletion mutations of  **mutS** ,  **mutL** , mutH, dam, polI, uvrD, mfd and recA were constructed in Hi strain Rd.  Ref: 11889052 EMBO J, 2002
- **MutS**  and  **MutL** , part of the post replication mismatch repair system, stimulate VSP repair.  Ref: 11585365 Mutat Res, 2001
- BACKGROUND The aim of this study was to search for mutations in the human  **mutS**  homolog 2 hMSH2 and human  **mutL**  homolog 1 hMLH1 genes in 25 unrelated Brazilian kindreds with suspected hereditary nonpolyposis colorectal cancer HNPCC.  Ref: 12095971 Ann Surg Oncol, 2002
- In vitro and in vivo studies of  **MutS** ,  **MutL**  and MutH mutants correlation of mismatch repair and DNA recombination.  Ref: 12606120 DNA Repair Amst , 2003
- Specific ternary complexes of  **MutS**   **MutL**  heteroduplexes were formed only during ATP hydrolysis.  Ref: 11888282 Biochemistry, 2002
- Until now, a number of works have focused on the function of  **MutS**  and  **MutL**  homologs during mismatch DNA repair.  Ref: 11553915 Mol Reprod Dev, 2001
- In order to understand the role of the yeast mismatch repair MMR system in the mutagenesis of stationary phase cells, we studied the effects of deletions in genes encoding  **MutS**  and  **MutL**  related proteins on the reversion frequency of the lys2 Delta Bgl frameshift mutation.  Ref: 12491007 Curr Genet, 2002
- Since the discovery of a link between the malfunction of post replicative MMR and human cancers, a number of works have focused on the function of  **MutS**  and  **MutL**  homologs in the correction of replication errors.  Ref: 12095912 Hum Mol Genet, 2002
- Whereas  **mutS**  and  **mutL**  genes are found in most organisms, the mutH gene is limited to some proteobacteria.  Ref: 11934505 FEMS Microbiol Lett, 2002
- The  **MutS**  homologous MSH and  **MutL**  homologous MLH proteins are highly conserved among all prokaryotes and eukaryotes.  Ref: 12582547 Theor Appl Genet< MedlineTA>Theor Appl Genet, 2002
- Concurrently, the frequency of spontaneous point mutations of  **mutS**  and  **mutL**  mutants from the slow strain was increased 20 to 30 fold to the level seen in the high strain.  Ref: 11359570 Mol Microbiol, 2001
- VSP repair and MMR share two proteins,  **MutS**  and  **MutL** , and although the two repair pathways are not equally dependent on these proteins, their dual use creates a competition within the cells between the repair processes.  Ref: 12067333 Mol Microbiol, 2002
- **MutL**  GFP formed foci in a subset of cells, and its localization was  **MutS**  dependent.  Ref: 11779496 Mol Cell, 2001
- **MutS**  and  **MutL**  homologs have been implicated in multiple genetic stabilization pathways.  Ref: 11717437 Proc Natl Acad Sci U S A, 2001
- These results implicate  **MutS**  alpha and  **MutL**  alpha in the suppression of gene amplification and suggest that defects in this genetic stabilization function may contribute to the cancer predisposition associated with mismatch repair deficiency.  Ref: 11717437 Proc Natl Acad Sci U S A, 2001
- We have adapted the Escherichia coli mismatch detection system, employing the factors  **MutS** ,  **MutL**  and MutH, for use in PCR based, automated, high throughput genotyping and mutation detection of genomic DNA.  Ref: 11222761 Nucleic Acids Res, 2001
- This phenotype is suppressed by a uvrD mutU defect, and partially suppressed by mutH,  **mutL** , or  **mutS**  mutations.  Ref: 11381137 Proc Natl Acad Sci U S A, 2001
- The DNA repair genes  **mutL**  and  **mutS**  were separated by the prophage remnant SF370.4.  Ref: 12441069 Virology, 2002
- the cause of MMR deficiency in these cell lines is due to a defective  **MutS**  alpha or  **MutL**  alpha.  Ref: 12173046 Oncogene, 2002
- The mammalian DNA mismatch repair genes belong to a family of genes that comprise several homologs of the Escherichia coli  **mutS**  and  **mutL**  genes.  Ref: 12114115 Trends Mol Med, 2002
- Evidence to support a role for the mismatch repair genes human  **mutL**  homolog 1 hMLH1 and human  **mutS**  homolog 2 hMSH2 in the etiology of colorectal cancer has come from linkage analysis, segregation studies, and molecular biologic analysis.  Ref: 12419761 Am J Epidemiol, 2002
